# Multi-Platform Compatible Software for Analysis of Polymer Bending Mechanics

**DOI:** 10.1371/journal.pone.0094766

**Published:** 2014-04-16

**Authors:** John S. Graham, Brannon R. McCullough, Hyeran Kang, W. Austin Elam, Wenxiang Cao, Enrique M. De La Cruz

**Affiliations:** Department of Molecular Biophysics and Biochemistry, Yale University, New Haven, Connecticut, United States of America; Politecnico di Milano, Italy

## Abstract

Cytoskeletal polymers play a fundamental role in the responses of cells to both external and internal stresses. Quantitative knowledge of the mechanical properties of those polymers is essential for developing predictive models of cell mechanics and mechano-sensing. Linear cytoskeletal polymers, such as actin filaments and microtubules, can grow to cellular length scales at which they behave as semiflexible polymers that undergo thermally-driven shape deformations. Bending deformations are often modeled using the wormlike chain model. A quantitative metric of a polymer's resistance to bending is the persistence length, the fundamental parameter of that model. A polymer's bending persistence length is extracted from its shape as visualized using various imaging techniques. However, the analysis methodologies required for determining the persistence length are often not readily within reach of most biological researchers or educators. Motivated by that limitation, we developed user-friendly, multi-platform compatible software to determine the bending persistence length from images of surface-adsorbed or freely fluctuating polymers. Three different types of analysis are available (cosine correlation, end-to-end and bending-mode analyses), allowing for rigorous cross-checking of analysis results. The software is freely available and we provide sample data of adsorbed and fluctuating filaments and expected analysis results for educational and tutorial purposes.

## Introduction

Biological systems must respond to mechanical stresses and strains in a manner that does not compromise their function. In some cases those responses also regulate cellular processes. For example, cytoskeletal polymers such as microtubules and actin filaments, provide cells with structural integrity and organization, generate forces that drive cell motility [1], [2], and play essential roles in cellular mechano-sensing [3]. To gain insight into how cytoskeletal filaments and higher-order structures, such as bundles and networks, provide mechanical responses and forces, it is necessary to quantify the intrinsic mechanical properties of individual filaments.

Cytoskeletal filaments display elastic properties between rigid rods and fully flexible polymers, and thus fall into the category of “semiflexible” polymers. The “wormlike chain” model is commonly used to describe semiflexible polymer mechanics and considers the end-to-end distance of a linear, unbranched polymer as a sum of unit vectors tangent to the polymer chain [4]. This model is characterized by a fundamental parameter [5], the bending persistence length (*L_p_*), that is extracted from analysis of the polymer's bending energy and the directional correlation of the unit tangent vectors.

The bending persistence length provides a useful quantitative measure of a polymer's bending rigidity. The *L_p_* is the segment length below which a polymer can be approximated as a rigid rod. Mathematically, *L_p_* is defined [4]:
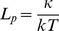
(1)where *κ* is the effective flexural rigidity of the polymer and *kT* is the thermal energy. We note that equation (1) does not consider contributions from coupling between deformations, such as twisting, that may contribute to the effective flexural rigidity [6].

Many investigations of polymer mechanics using the wormlike chain model involve specialized measurement and analysis techniques that are often not readily within reach of most educators or biological researchers. Further, to our knowledge, there is no commercially available software package for performing analyses of polymer bending mechanics. Motivated by those facts, we developed software, *Persistence*, to determine the bending *L_p_* of biopolymers from images of surface-adsorbed or freely fluctuating macromolecules. Those images can be obtained from atomic force microscopy, electron microscopy, fluorescence imaging or any other available imaging method.

To foster the broadest possible accessibility, we created a user-friendly graphical interface to facilitate entry of initial variable values and analysis commands, and made it multi-platform compatible and freely available (see File S1). Sample data for tutorial or educational use is included with the software.

## Description of *Persistence* Software

### Pre-Processing

For *Persistence* to function effectively, images of the filaments to be analyzed must be processed to remove noise and enhance the prominence of filament shapes. ImageJ [7] can be used for that purpose, although any other image processing software should work just as well. A general algorithm includes five steps: background subtraction, smoothing, contrast enhancement, thresholding and skeletonization. Background subtraction and smoothing reduce the noise levels in the image. Smoothing is particularly important for noisy images as it effectively averages out background noise to a low enough level that it will not interfere with the skeletonization and final analysis. Many filtering methods are available, depending on the image processing software. For our analyses of actin filaments, a Gaussian blur filter has worked best, but that may not always be the case.

**Figure 1:**
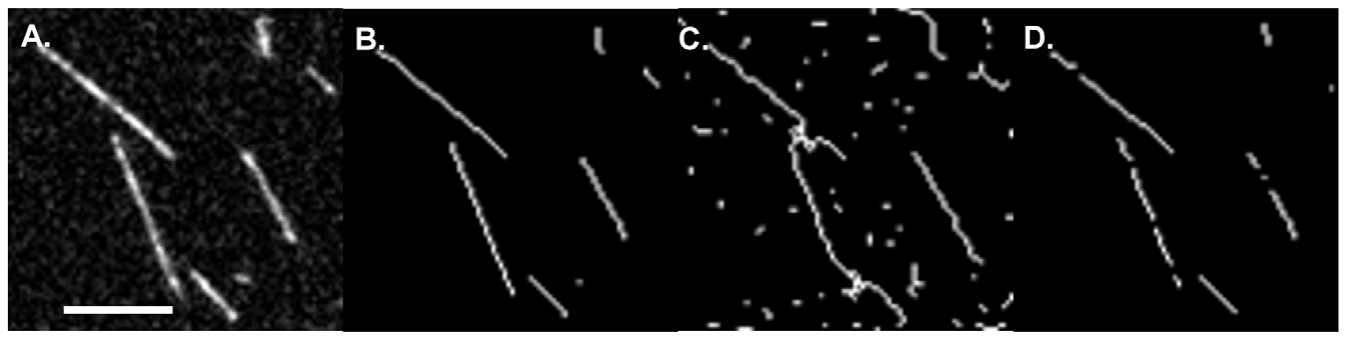
The effect of thresholding on filament skeletonization. **A**. Original image. **B**. Image skeletonized with proper thresholding. **C**. Image with superfluous branches due to improper thresholding. In this case the threshold is too high. **D**. Image with fragmented filaments due to improper thresholding in the opposite extreme from panel C, i.e. threshold set too low. Scale bar is 3 µm.

**Figure 2:**
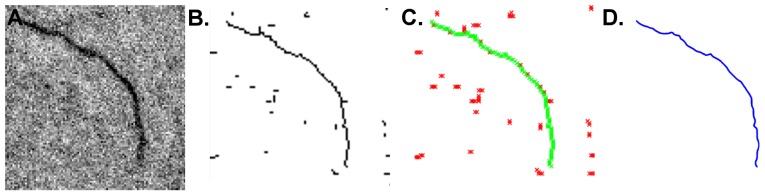
Digital image processing for filament shape reconstruction. **A**. A representative image of a fluorescently-labeled actin filament. **B**. The image after thresholding, and skeletonization. **C**. Result of detection and tracking algorithms. Note absence of artifactual branching. **D**. Final result of filament shape reconstruction.

The final three adjustments (contrast enhancement, thresholding and skeletonization) enhance prominence of filament shapes so that the software can efficiently track them (Figures 1 and 2A & B). Contrast enhancement optimizes the signal-to-noise ratio and normalizes the signal for processing and visualization. Thresholding enhances the background subtraction by limiting pixel values to a selected range. Care should be taken when adjusting threshold values to ensure that artificial gaps or branches are minimized (Figure 1). Skeletonization does two things to the image. First, it converts the image to a binary format so that pixel values are either 0 or 255 for an RGB image, effectively on (255) or off (0). For that reason, it is especially important that as much “noise” as possible be removed from the image. Second, skeletonization pares down the filament shapes to a single pixel width so that they can be easily tracked by the tracking algorithm in the *Persistence* software. Throughout pre-processing, it is important to compare the processed image with the original after each processing step. Such crosschecking allows for immediate correction of processing artifacts that may arise, such as the artificial gaps or branches mentioned above.

### The Graphical User Interface

#### Layout

**Figure 3:**
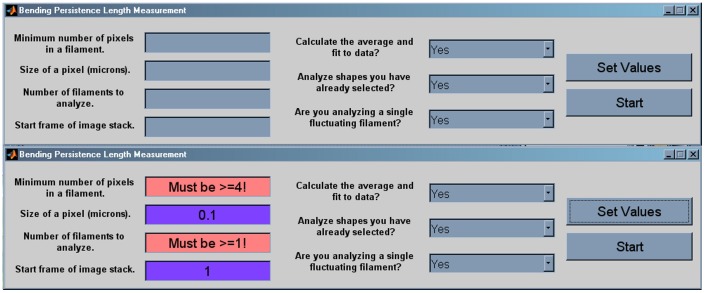
The graphical user interface (GUI). At top is a screen shot of the GUI upon opening. On the left are fields for entry of initial variable values, in the center are the pull-down response fields and on the right are the control buttons. After variable and pull-down values are selected, the “Set Values” button is depressed and chosen variable values are highlighted in purple, or an error is reported. At bottom is the GUI showing data entered with errors resulting from invalid values.

The function of the graphical user interface (GUI) is to allow for easy entry of variables and instructions necessary for subsequent analysis. Numerical variables are defined in the column of boxes on the left side of the GUI (Figure 3A). When the values are set, the boxes report either the entered variable values or an error if the value is outside parameter limits (Figure 3B). In the center of the GUI are yes/no questions to be answered based on the type of analysis desired, and on the right are buttons to confirm the values entered and to start the analysis.

#### Variable definitions

Four variables must be set prior to analysis:

a. “*Minimum number of pixels in a filament*.” is the minimum length of the filament/polymer and must be at least 4.

b. “*Size of a pixel*.” is the size, in microns, of a single camera pixel under the magnification of the instrument.

c. “*Number of filaments to analyze*.” is the number of filaments to be used for analysis. The program searches for filament ends beginning at the bottom left of the image, reading each row of pixels from left to right, proceeding from bottom to top. To include all filaments in an image stack, enter *inf* (∞). The analysis time depends on the total number of filaments. The potential exists to overload memory available for the program if too many filaments are selected. We recommend starting with 100 to 200 filaments.

d. “*Start frame of image stack*.” is the frame number of the image stack on which to begin loading images into the software. This is useful for the serial analysis of a large image stack. The program can be aborted during filament selection and all images selected up to that time will be used for analysis.

#### Preliminary processing instructions

a. “*Calculate the average and fit to data*?” should be set to “Yes” if a final cosine correlation fit is desired. The fit generated by *Persistence* is a reasonable estimate of the results. However, independent analysis should be performed with a nonlinear regression program.

b. “*Analyze shapes you have already selected*?” should be set to “Yes” if filament shapes previously selected from an image stack will be reanalyzed using the same input parameters. This feature was implemented to prevent the user from having to reselect filament shapes in the event of a computer crash while calculating information from the filament shapes already selected (data is *not* saved if the crash occurs during the selection process).

c. “*Are you analyzing a single fluctuating filament*?” should be set to “Yes” if a single filament fluctuating freely in solution will be analyzed.

#### Initialization of analysis

Once the variable and preliminary instructions are entered in the GUI, the chosen values are set in the software by clicking the “Set Values” button. The numerical variable column either confirms the values entered or presents an error message (Figure 3B). If there is an error message, that variable value must be changed so that it is within the allowed parameters. As long as the value entered is numerical, the only possible errors are in the “*Minimum number of pixels*…” variable which must be ≥4, or the “*Number of filaments*…” variable which must be ≥1. Importantly, the software cannot determine if the entered values are reasonable. For example, a value of 1000 µm entered for “*Size of a pixel*.” will be accepted, but it is clearly incorrect. Once the software has confirmed that no errors exist, the analysis is initiated by clicking the “Start” button.

## Steps in the Analysis Process

### Filament reconstruction

The first step in the analysis is to reconstruct the filament shapes from the skeletonized images. This step begins by locating the filament ends then applying a directional tracking algorithm beginning at one of the ends. The filament-tracking algorithm avoids artifactual branching of the skeleton and tracks over gaps of one or two pixels. An average third-order Bézier spline is created along the pixels that represent the filament backbone. The continuous spline allows for a more fine-grained shape reconstruction, essentially eliminating the effect of pixelation introduced in the skeletonization procedure and increasing the number of tangent vectors along a filament (Figure 2 C & D) [8]. Filament reconstruction may give erroneous results if selected filaments are too close together. In such cases, the reconstruction may join two separate filaments together and analyze them as one (Figure S1 in File S1).

### Analysis methods

Filaments can be analyzed using three methods: cosine correlation, end-to-end distance analysis and bending mode analysis. The first two methods can be used for all filament analyses while bending mode analysis is used only for analysis of a single fluctuating filament.

a. Cosine correlation

The first analysis method is an angular analysis that determines the persistence length (*L_p_*) from the two-dimensional cosine correlation (*C*) of tangent angles *θ* along a segment of length *s* using the fitting function:
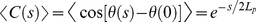
(2)


That function determines *L_p_* from two-dimensional shape fluctuations [8], [9]. The angular correlation is determined with respect to an initial reference angle (*θ*(0) in Eq. 2). The argument of the cosine function, *θ*(s)-*θ*(0), is subject to experimental and computational uncertainty given resolution and pixel size limitations. Because of the symmetry of the cosine function, ±(*θ*(s)-*θ*(0)) have identical cosine values and the uncertainty in determination of the relative angles will asymmetrically propagate to the final cosine value. Therefore, the resultant cosine value will be lower than the actual value (Figure S2 in File S1). This uncertainty can be accounted for by fitting data to an exponential, 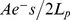, without constraining the decay amplitude or correlation to unity when the segment length approaches zero. For freely fluctuating filaments, measures must be taken to ensure that fluctuations are constrained to two dimensions [10].

b. End-to-end distance analysis

The second analysis fits the mean square end-to-end distance (<*R^2^*>) as a function of the contour length (*L*) to the equation [11], [12],
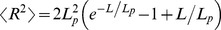
(3)from which *L_p_* can be directly extracted. This method is less accurate than the cosine correlation analysis, but serves as a useful verification. However, since this method considers only the global geometry of an individual filament, each filament analyzed represents a single data point. Therefore, end-to-end analysis requires a larger number of filaments than cosine correlation analysis to obtain a statistically significant sample.

c. Bending mode analysis

The additional analysis for single fluctuating filaments determines the angular bending modes of a filament determined through Fourier analysis of the filament shape. The amplitudes (*a*) of the bending modes (*a_n_*, where *n* is the bending mode number 1, 2, 3…10) are used to calculate the persistence length using [9]:
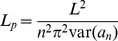
(4)


## Effect of Initial Input on Results

*Persistence* requires a minimal number of input parameters to function properly. Those parameters (shown in Figure 3) are customizable; facilitating its use for diverse applications. The impact of those parameters on the collection and analysis of data is straight-forward but critical for obtaining meaningful results. In particular, two variable values have a crucial impact on processing and the final results; pixel threshold (*Minimum number of pixels in a filament*.) and pixel size (*Size of a pixel*.).

**Figure 4:**
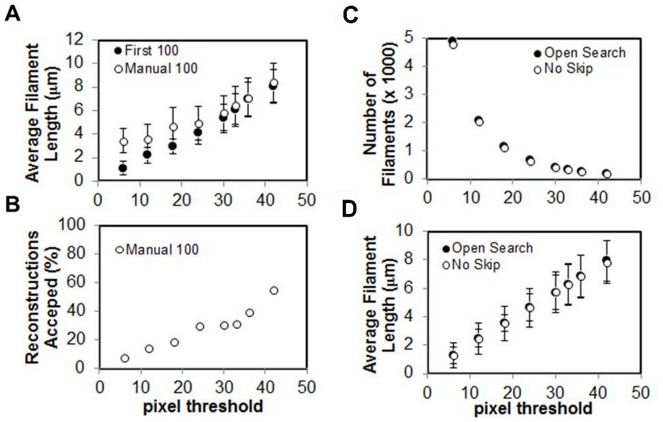
Comparison of manual and automatic filament selection options. **A**. Plot of average filament length as a function of pixel threshold (“*Minimum number of pixels*…”) setting comparing automatic (solid circles) and manually (open circles) accepted filament reconstructions. **B**. Percent of filaments manually accepted as a function of the pixel threshold setting. **C**. Number of filaments reconstructed using the pixel gap function (solid circles) and without pixel skipping (open circles) as a function of the pixel threshold setting. **D**. Average filament length for the two cases in panel C showing no significant difference between them.

“*Minimum number of pixels*…” defines the minimum number of contiguous pixels required for polymer reconstruction. The minimum allowed value of “4” was chosen because a polymer spanning fewer than four contiguous pixels will usually display little to no detectable curvature. Care must be taken when choosing a value for “*Minimum number of pixels*…” since, as the value is increased above the minimum allowed, shorter polymers will no longer be reconstructed and the average polymer length will increase (Figure 4A, for bare actin filaments). This trend holds whether or not the polymer reconstructions are manually approved by the user (Figure 4A). Note that when the “*Minimum number of pixels*…” value is low, if polymer reconstructions are not manually verified by the user, it is then possible for poorly resolved polymer fragments and/or background noise to contribute to the data, resulting in low average polymer length (Figure 4A). A properly chosen value for “*Minimum number of pixels*…” promotes recognition of polymers while minimizing the number of false polymer reconstructions that may occur if the image has a low signal-noise ratio. Particularly at low pixel thresholds, many polymer reconstructions may contain contributions from noise or otherwise misrepresent polymers in the image. In that case, the user may manually screen the reconstructions (1267 polymers reconstructed to get 100 approved, ∼8% success, see Figure 4B). Increasing the pixel threshold increases probability that a reconstruction corresponds to a polymer (182 polymers reconstructed to get 100 approved, ∼60% success, see Figure 4B), eliminating the tedious process of manually screening many erroneous reconstructions.

“*Size of a pixel*.” is the size of a camera pixel under the magnification of the instrument which will vary depending on the microscope and camera set-up employed to capture the images. The “*Size of a pixel*.” value directly affects characterization of polymers by the *Persistence* algorithm in that the size of the image pixels (in microns) is critical for the program to accurately calculate polymer properties such as contour length, end-to-end distance and the persistence length (*L_p_*). Users must be careful when entering a value for “*Size of a pixel*.” as there are no limits set in the software.

## Handling Pixel Gaps

During the reconstruction process, the *Persistence* software uses a tracking algorithm that examines all pixels in a two-pixel radius to determine the direction the reconstruction should progress while skipping small gaps and avoiding termination at artificial branches. The tracking algorithm skips over one black pixel in a polymer and continues reconstruction, rather than interpreting the gap as the polymer end. For bare actin filaments adsorbed to a poly-L-lysine treated glass surface, the ability of the search method to skip pixels has negligible (<5%) impact on the number of filaments reconstructed at any pixel threshold (Figure 4C), and does not appear to increase the average length of actin filaments (Figure 4D). However, the ability to skip pixels during reconstruction of polymers is crucial for more flexible polymers that undergo out-of-plane conformational fluctuations or for samples labeled with a dye that rapidly photobleaches.

The polymer tracking algorithm was incorporated to account for false branches or gaps in actin filaments that arise as artifacts of pre-processing, as described above. Such artifacts arise from background subtraction, smoothing and, in particular, thresholding. Another major contributor to pre-processing artifacts is the method of sample preparation. With diligence, all of these contributions to undesirable artifacts can be minimized.

Filament ends are recognized by locating a point with only one or two nearest neighbors. The algorithm searches for either a single, nonzero pixel within two pixels of the starting point or two nonzero pixels, lying side-by-side, in one direction from the starting point, to account for filaments running diagonally. The user has the option to manually select filament ends for detection or to allow the software to perform the search. Manual selection is recommended in cases of high filament density, which could complicate automatic identification of filament ends. However, careful sample preparation and pre-processing reduces the need for subjective user input. In addition to the difficulty of locating filament ends during reconstruction, the pixel gap function can adversely influence filament size analysis, particularly when substantial pre-processing artifacts are present. For example, a gap of more than two pixels in a single filament will treat a single filament as two filaments, reducing the measured average filament length.

## Analyzing a Single Fluctuating Filament

The *Persistence* program can be used to analyze a single fluctuating filament in two dimensions. The sample chamber should be prepared to allow thermal fluctuations while restricting them to two dimensions. It is important that both ends of the filament be in focus at all times since any blurred pixels at the ends will affect filament reconstruction in a way that will significantly affect the final *L_p_* value. One then acquires a stack of time lapse images of the filament. The persistence length of fluctuating filaments is determined through discrete Fourier analysis (eq. 4). Single fluctuating filaments can also be analyzed using cosine correlation (eq. 2) and/or end-to-end analysis (eq. 3).

**Figure 5:**
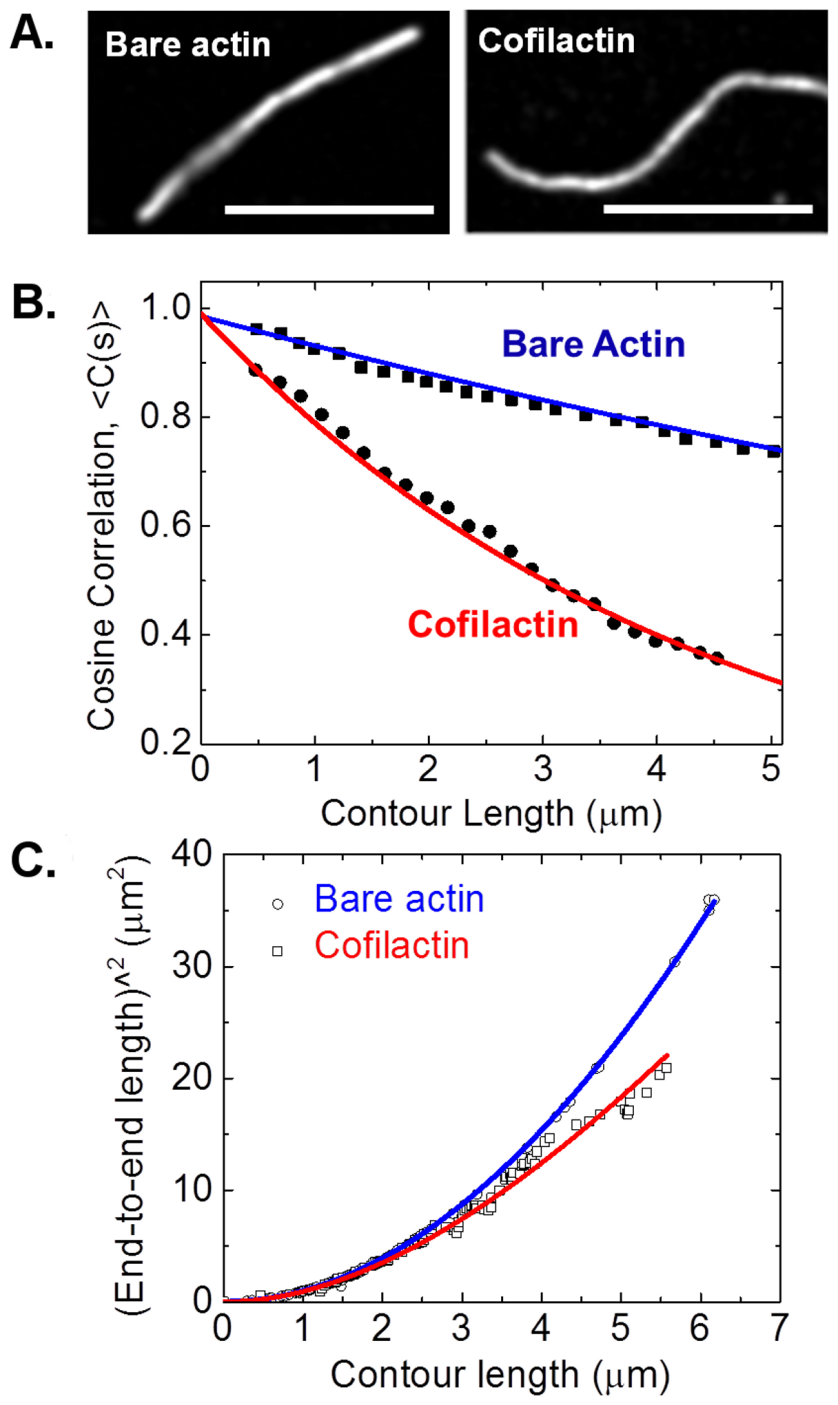
Determination of actin filament bending persistence lengths. **A**. Representative fluorescence images of bare actin and cofilactin filaments. Cofilin binding density is >0.9. Scale bars are 5 µm. **B**. Cosine correlation data of bare actin (filled squares) and cofilactin filaments (filled circles) (N = 300 filaments). Solid lines represent best fits to equation 2 (cosine correlation) yielding L_p_ = 8.8±0.9 µm, L_p_ = 2.8±0.6 µm, for bare actin filaments and cofilactin filaments, respectively. **C**. End-to-end analysis data of bare and cofilactin filaments. Solid lines represent best fits to equation 3, L_p_ = 10.8±1.8 µm, L_p_ = 3.8±1.2 µm, for bare actin filaments and cofilactin filaments, respectively.

The best candidates for analysis using the *Persistence* program are semi-flexible biopolymers in which the contour length is larger than, but within an order of magnitude of, the persistence length. For example, bare actin filaments and actin filaments decorated with the actin binding protein cofilin (cofilactin filaments) undergoing thermally driven shape fluctuations in two dimensions are two excellent examples (Figure 5). When compared on the same length scale, cofilactin filaments have greater bending flexibility than bare actin filaments (Figure 5A) and, therefore, a lower bending persistence length. That result holds for both the cosine correlation analysis (Figure 5B) and end-to-end analysis (Figure 5C).

## Conclusion

The software described was developed primarily for researchers with limited experience in analysis of polymer bending mechanics. Accordingly, it is also well suited for educational purposes. Those with extensive polymer physics/chemistry backgrounds may also benefit by saving the time required to develop the software on their own. Although our discussion is limited to protein biopolymers, the software can be used to analyze any polymer chain visualized either when adsorbed to a substrate or while fluctuating freely in solution.

Persistence was coded in MatLab and is freely available at http://medicine.yale.edu/lab/edelacruz/index.aspx. While MatLab is not required to run Persistence, the free MatLab MCR (http://www.mathworks.com/products/compiler/mcr/) must be downloaded and installed in the absence of MatLab. Sample data of adsorbed and fluctuating filaments and expected analysis results are provided for educational and tutorial purposes. Both raw and pre-processed data are included to accommodate flexibility in the depth of instruction desired.

We will maintain the software to address any deficiencies or bugs and expand its functionality as research or educational demands require. Users are encouraged to submit recommendations to EMDLC.

## Supporting Information

File S1 **Text contains notes on sample preparation, analysis results for included sample data and descriptions of output files generated by** ***Persistence*** **.** Three figures are also included as described below. **Figure S1** Potential error in filament reconstruction. **A**. An image of two actin filaments that cross each other. **B**. A correct filament reconstruction from the image in **A** despite filament crossover. **C**. An image of the same two actin filaments in **A** at a subsequent time. **D**. An incorrect filament reconstruction from the image in **C** showing a continuation of the filament reconstruction along the adjacent filament. **Figure S2** Effect of standard deviation of relative angle determination on cosine correlation value of a straight line. Top panel shows relative tangent angle as a function of segment length *s*, with 0 (black square), 0.1 (red circle), 0.25 (green triangle) and 0.35 (blue inverted triangle) radians of standard deviation. Bottom panel shows the corresponding cosine values. The average cosine values corresponding to standard deviations of 0, 0.1, 0.25 and 0.35 radians in relative angle determination are 1, 0.995, 0.97 and 0.94 respectively. The data shows that the non-linear and non one-to-one relationship between an angle and its cosine leads to asymmetric error propagation in the cosine value resulting from the error in the determination of relative angles. Since the cosine function cannot have a value greater than one, the asymmetric error propagation will reduce the cosine value. **Figure S3** Cosine correlation plot. The semiflexible regime ranges approximately from 0.5*L_p_ to 8*L_p_. For polymers having *L*>*L_p_* and an instrument with high enough resolution, a polymer segment can be chosen such that *L_s_*∼*L_p_* to run the analysis.
(DOCX)
