# Supplementary material for: Multi-Platform Compatible Software for Analysis of Polymer Bending Mechanics
Source: PLoS One. 2014 Apr 16;9(4):e94766. doi: 10.1371/journal.pone.0094766 (PMC3989245; doi:10.1371/journal.pone.0094766)
Supplement: File S1 — Text contains notes on sample preparation, analysis results for included sample data and descriptions of output files generated by Persistence . Three figures are also included as described below. Figure S1 Potential error in filament reconstruction. A. An image of two actin filaments that cross each other. B. A correct filament reconstruction from the image in A despite filament crossover. C. An image of the same two actin filaments in A at a subsequent time. D. An incorrect filament reconstruction from the image in C showing a continuation of the filament reconstruction along the adjacent filament. Figure S2 Effect of standard deviation of relative angle determination on cosine correlation value of a straight line. Top panel shows relative tangent angle as a function of segment length s, with 0 (black square), 0.1 (red circle), 0.25 (green triangle) and 0.35 (blue inverted triangle) radians of standard deviation. Bottom panel shows the corresponding cosine values. The average cosine values corresponding to standard deviations of 0, 0.1, 0.25 and 0.35 radians in relative angle determination are 1, 0.995, 0.97 and 0.94 respectively. The data shows that the non-linear and non one-to-one relationship between an angle and its cosine leads to asymmetric error propagation in the cosine value resulting from the error in the determination of relative angles. Since the cosine function cannot have a value greater than one, the asymmetric error propagation will reduce the cosine value. Figure S3 Cosine correlation plot. The semiflexible regime ranges approximately from 0.5*Lp to 8*Lp. For polymers having L>Lp and an instrument with high enough resolution, a polymer segment can be chosen such that Ls∼Lp to run the analysis. (DOCX) [file pone.0094766.s001.docx]

**SUPPORTING INFORMATION for Multi-Platform Compatible Software for Analysis of Polymer Bending Mechanics**

**Notes on Sample Preparation**

We include here brief notes on final sample preparation techniques used for the actin filament data displayed in the figures as well as in the sample data. Notes on general sample preparation considerations are also included.

Adsorbed filaments: Fluorescently-labeled actin filaments (Alexa-labeled with labeling efficiency of ~70 %) were immobilized on poly-L-lysine (Sigma) treated coverslips [10,14] [15].

Fluctuating filaments: Slides were treated with bovine serum albumin (BSA) to minimize adsorptive interactions with the glass surfaces. To ensure quasi-2D motion, the depth of the sample was < 3 µm to limit axial rotation of the filament [10,15].

*Considerations for General Sample Preparation*

Reliable determination of a polymer’s *L_p_* value relies on sample preparation, visualization techniques (e.g. electron microscopy, atomic force microscopy etc.) and their requisite modifications. For example, when using fluorescence visualization, care must be taken that the fluorescence probe doesn’t affect the mechanical properties of the polymer. Fluorescent dyes can affect the mechanical properties of DNA at high dye to DNA base ratios [13]. Similarly, fluorescent phallotoxins used to visualize actin filaments stiffen them [8]. Conceivably, the effect could transfer to other polymers.

One major source of artifacts affecting analysis is a “dirty” sample. In the case of actin, there may be many very short filaments and unpolymerized subunits throughout the sample which, for the purpose of the analysis discussed here, are considered contaminants. If too numerous, those contaminants can make background subtraction extremely difficult, if not impossible, and interfere with single filament reconstruction. Even in small amounts, sample contaminants can introduce errors in thresholding and smoothing. Such contaminants can often be reduced in number by a thorough rinsing of the sample after adsorption to the substrate prior to imaging. An important caveat is that sample rinsing will increase the filament size distribution due to elimination of many shorter filaments. Such a shift will likely affect average length measurements, but is not a concern when measuring the persistence length. In general, for any experimental method, the polymers should be confined to two dimensions, be at low enough concentration so that polymers in the image are isolated and have a contour length where adequate bending can be observed (*i.e. L*_p_  ~ *L*; Figure S3, below).

The polymer length, *L*, is of critical importance for accurate determination of *L_p_*. Semiflexible polymers, for which *L* ~ *L_p_*, are ideal candidates for analysis (Figure S3, below). In the rigid rod regime for which *L* < *L_p_*, a polymer will appear straight with no detectable loss in tangent angle correlation (Eq. 2), resulting in a lack of information needed to determine *L_p_*. In the opposite extreme, the fully flexible regime for which *L* >> *L_p_*, there will complete loss of tangent angle correlations, again resulting in too little information for determination of *L_p_*. However, if the image resolution is high enough (such as in electron microscopy data for DNA), the *L_p_* of fully flexible polymers can be determined by selecting a segment length, *L_s_*, such that *L_s_* ~ *L_p_*. We recommend no fewer than 4 pixels per *L_p_* as the minimum image resolution.

**Analysis Results for Included Sample Data**

Adsorbed filaments (0.18 µm/pixel): Cosine correlation analysis yields L_p_ of 14.3 (± 0.85) µm. End-to-end analysis yields L_p_ of 18.00 (± 1.0) µm, for 200 filaments selected.

Fluctuating filaments (0.18 µm/pixel): From cosine correlation analysis L_p_ = 5.10 ± 0.58 µm

Descriptions of generated output files:

xxx_selections: used for reanalyzing previously selected filaments.

xxx_Nfils: raw cosine correlation values

xxx_tan_stdev: segment length (1^st^ column), averaged cosine correlation values (2^nd^ column), standard error (3^rd^ column)

xxx_bending modes: frame number (1^st^ column), contour length (2^nd^ column), end to end length (3^rd^ column), centroid (4^th^-5^th^ columns), first 10 mode amplitudes (6^th^- 15^th^ columns)

xxx_end_to_end_vs_contour_lengths: contour length (1^st^ column) end-to-end length (2^nd^ column)

xxx_NDist: Distribution of independent points N

xxx_tangent_angles: raw data for tangential angles

xxx_contours: raw data for contour lengths

Note: “xxx” in the above represents the user-defined file name.

| 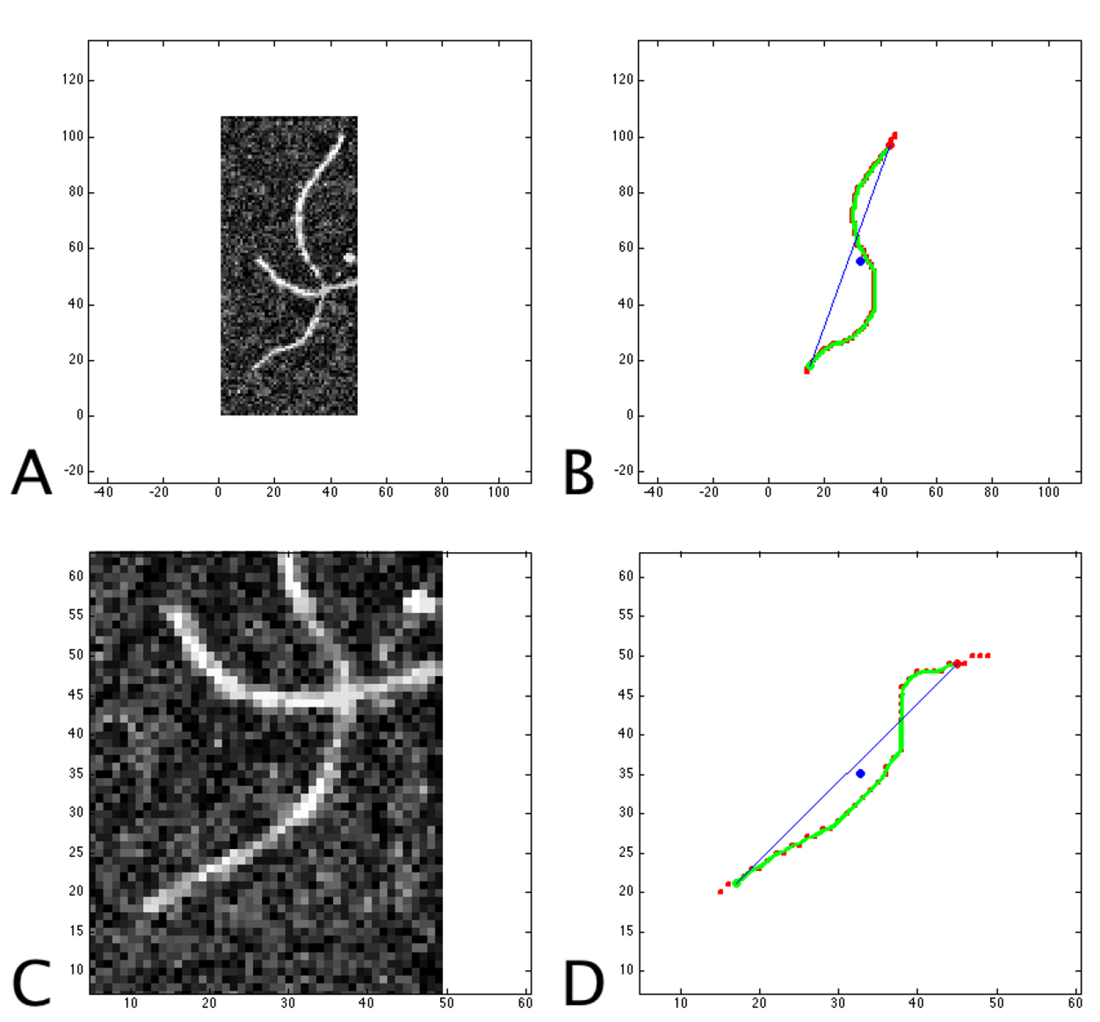 |
| --- |
| **Figure S1.** Potential error in filament reconstruction. **A.** An image of two actin filaments that cross each other. **B.** A correct filament reconstruction from the image in **A** despite filament crossover. **C.** An image of the same two actin filaments in **A** at a subsequent time. **D.** An incorrect filament reconstruction from the image in **C** showing a continuation of the filament reconstruction along the adjacent filament. |

| 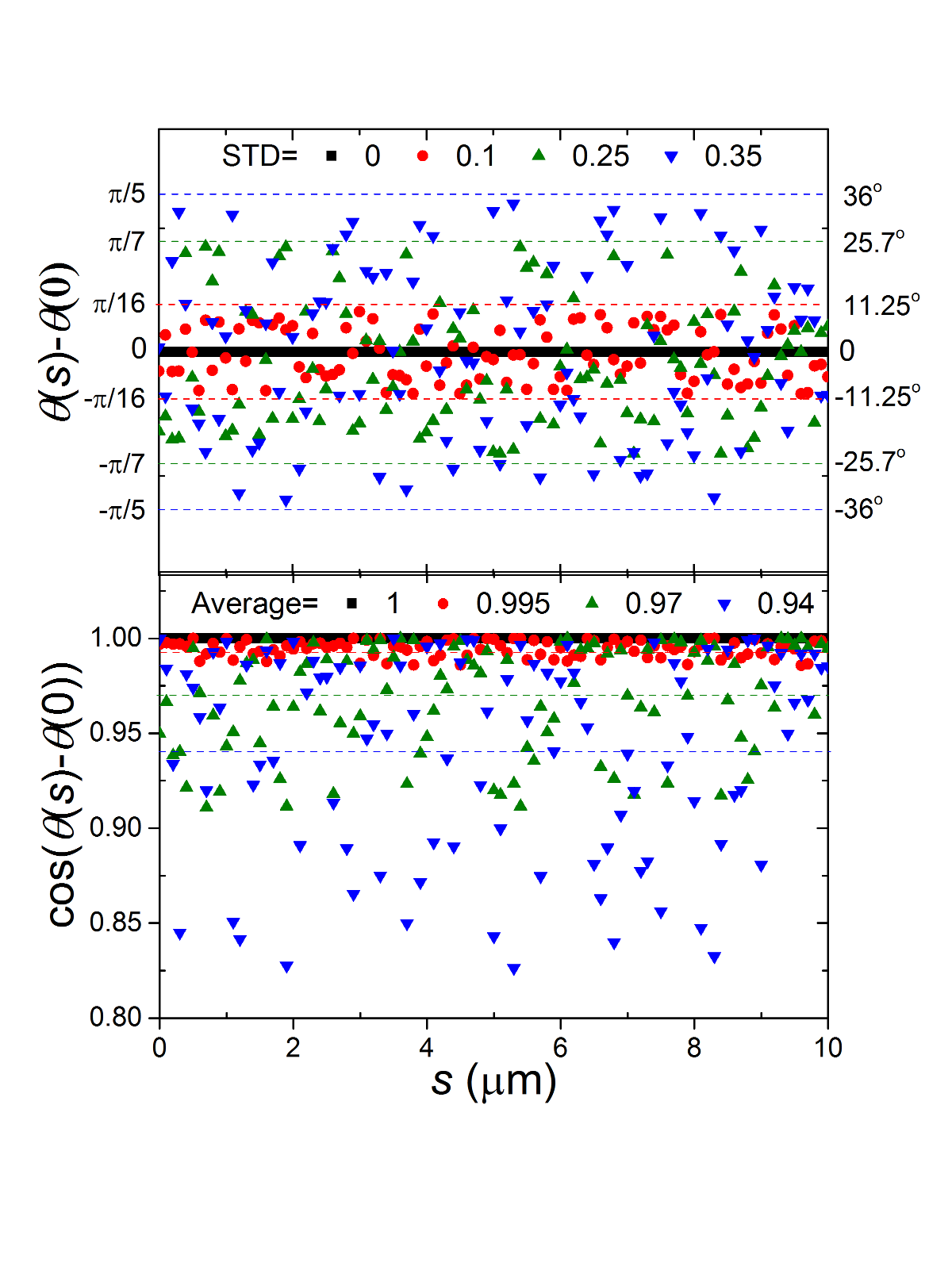 |
| --- |
| **Figure S2.** Effect of standard deviation of relative angle determination on cosine correlation value of a straight line. Top panel shows relative tangent angle as a function of segment length *s*, with 0 (black square), 0.1 (red circle), 0.25 (green triangle) and 0.35 (blue inverted triangle) radians of standard deviation. Bottom panel shows the corresponding cosine values. The average cosine values corresponding to standard deviations of 0, 0.1, 0.25 and 0.35 radians in relative angle determination are 1, 0.995, 0.97 and 0.94 respectively. The data shows that the non-linear and non one-to-one relationship between an angle and its cosine leads to asymmetric error propagation in the cosine value resulting from the error in the determination of relative angles. Since the cosine function cannot have a value greater than one, the asymmetric error propagation will reduce the cosine value. |

| 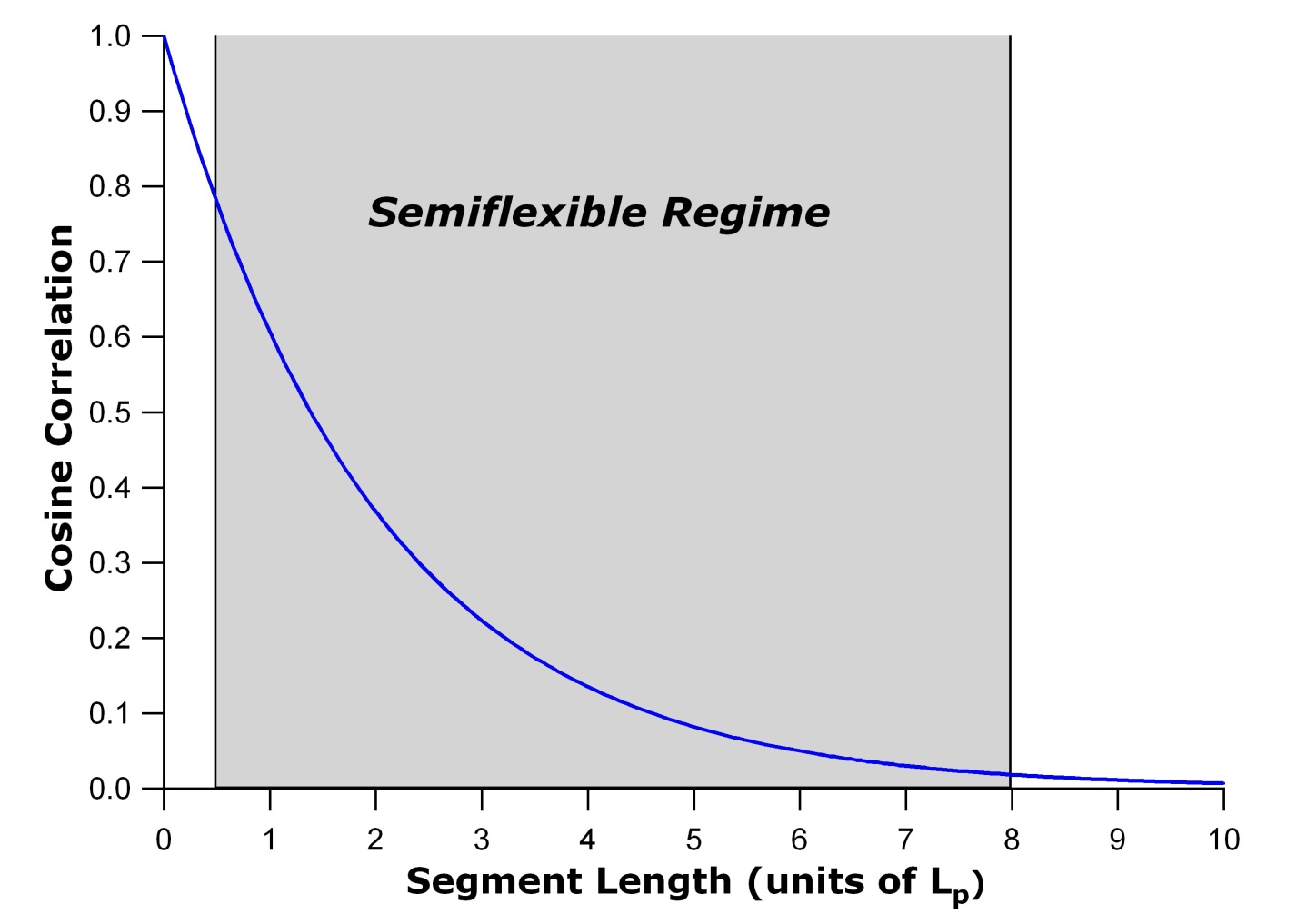 |
| --- |
| **Figure S3.** Cosine correlation plot. The semiflexible regime ranges approximately from 0.5**L_p_* to 8**L_p_*. For polymers having *L* > *L_p_* and an instrument with high enough resolution, a polymer segment can be chosen such that *L_s_* ~ *L_p_* to run the analysis. |
